# Caffeine‐Operated Synthetic Modules for Chemogenetic Control of Protein Activities by Life Style

**DOI:** 10.1002/advs.202002148

**Published:** 2020-12-13

**Authors:** Tianlu Wang, Lian He, Ji Jing, Tien‐Hung Lan, Tingting Hong, Fen Wang, Yun Huang, Guolin Ma, Yubin Zhou

**Affiliations:** ^1^ Center for Translational Cancer Research Institute of Biosciences and Technology Texas A&M University Houston TX 77030 USA; ^2^ Center for Epigenetics and Disease Prevention Institute of Biosciences and Technology Texas A&M University Houston TX 77030 USA; ^3^ Department of Translational Medical Sciences College of Medicine Texas A&M University Houston TX 77030 USA

**Keywords:** allosteric switch, caffeine, chemical biology, chemically induced dimerization, nanobody, SARS‐Cov‐2

## Abstract

A genetically encoded caffeine‐operated synthetic module (COSMO) is introduced herein as a robust chemically induced dimerization (CID) system. COSMO enables chemogenetic manipulation of biological processes by caffeine and its metabolites, as well as caffeinated beverages, including coffee, tea, soda, and energy drinks. This CID tool, evolved from an anti‐caffeine nanobody via cell‐based high‐throughput screening, permits caffeine‐inducible gating of calcium channels, tumor killing via necroptosis, growth factors‐independent activation of tyrosine receptor kinase signaling, and enhancement of nanobody‐mediated antigen recognition for the severe acute respiratory distress coronavirus 2 (SARS‐CoV‐2) spike protein. Further rationalized engineering of COSMO leads to 34–217‐fold enhancement in caffeine sensitivity (EC_50_ = 16.9 nanomolar), which makes it among the most potent CID systems like the FK506 binding protein (FKBP)–FKBP rapamycin binding domain (FRB)–rapamycin complex. Furthermore, bivalent COSMO (biCOMSO) connected with a long linker favors intramolecular dimerization and acts as a versatile precision switch when inserted in host proteins to achieve tailored function. Given the modularity and high transferability of COMSO and biCOSMO, these chemical biology tools are anticipated to greatly accelerate the development of therapeutic cells and biologics that can be switched on and off by caffeinated beverages commonly consumed in the daily life.

## Graphical abstract

A robust chemically induced dimerization (CID) system, caffeine‐operated synthetic module (COSMO), is developed for chemogenetic control of various protein activities in live cells. Bivalent COSMOs with various linkers are engineered to enhance the caffeine sensitivity toward the low nanomolar range, and to enable intramolecular dimerization for precise control of protein functions with caffeine and its metabolites, as well as caffeinated beverages.

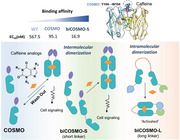

## 1 Introduction

The chemically induced dimerization (CID) technique has been widely applied to control biological processes via homo‐ and heterodimerization of engineered targets.^[^ ^1^ ^]^ The most popular CID system takes advantage of rapamycin or its analogs to dimerize FKBP12 and FRB domains of mammalian target of rapamycin (mTOR).^[^ ^2^ ^]^ However, the conventional FK506 binding protein (FKBP)–FKBP rapamycin binding domain (FRB) CID system is fraught with perturbation to endogenous mTOR signaling, which hampers its applications under certain biological contexts.^[^ ^3^ ^]^ The use of rapamycin is further limited due to its irreversibility on short timescales and chemical complexity.^[^ ^4^ ^]^ The development of rapalogs (such as AP21967,^[^ ^5^ ^]^ iRap,^[^ ^6^ ^]^ and cRap^[^ ^7^ ^]^) partially mitigates the concern of perturbation to host physiology but may require higher doses of expensive chemical dimerizers with narrow safety profiles.^[^ ^8^ ^]^ Recently, anti‐caffeine heavy‐chain‐only antibody (acVHH) emerges as a potential CID system by enabling protein homodimerization in the presence of caffeine.^[^ ^9^ ^]^ This caffeine‐operated CID system offers a more economic and safer solution, considering that caffeine is nontoxic, cheap, readily available, and exhibits no overt side effects at doses ((1–10) × 10^−6^ m) comparable to the average caffeine consumption in human's daily life.^[^ ^10^ ^]^ However, the relatively weak binding affinity of wild‐type (WT) acVHH impedes its further uses in many biological studies when compared to the FKBP–FRB system (*K*_d_ = (500–600) × 10^−9^ m for caffeine vs (10–20) × 10^−9^ m with rapamycin).^[^ ^11^, ^12^ ^]^ Moreover, extensive engineering and applications of the FKBP–FRB‐based CID system (RapR, UniRapR, and rapamycin‐regulated targeted activation of proteins (RapR‐TAP) ) have been reported,^[^ ^13^ ^]^ but the potential of the acVHH‐based CID system remains largely untapped.

Herein, we report a caffeine‐operated synthetic module (COSMO) after engineering acVHH with varying EC_50_ values for caffeine and its major metabolites via site‐directed mutagenesis and optimization of its bivalent forms by taking advantage of a high‐throughput cell‐based cytosol‐to‐plasma membrane (PM) translocation assay. We demonstrate that two copies of COSMO bearing the Y104W mutation connected with a short rigid linker (biCOSMO‐S) tend to form an intermolecular dimer with a higher affinity toward caffeine (EC_50_ = 16.9 × 10^−9^ m). By contrast, the bivalent COSMO with a long linker (biCOSMO‐L) functions as an intramolecular dimerizer, thereby permitting caffeine‐controllable conformational switch after insertion into a host protein—much like uniRapR does upon rapamycin treatment.^[^ ^14^ ^]^ We further showcase the application of COSMO to remotely control protein subcellular localization, ion channel gating, necroptotic cell death, tyrosine‐receptor‐kinase‐mediated cell signaling, viral antigen–nanobody interactions, and actin cytoskeleton remodeling by using caffeine or caffeinated beverages such as coffee and soda.

## 2 Results

### 2.1 Characterization and Optimization of COSMO

**Figure 1:**
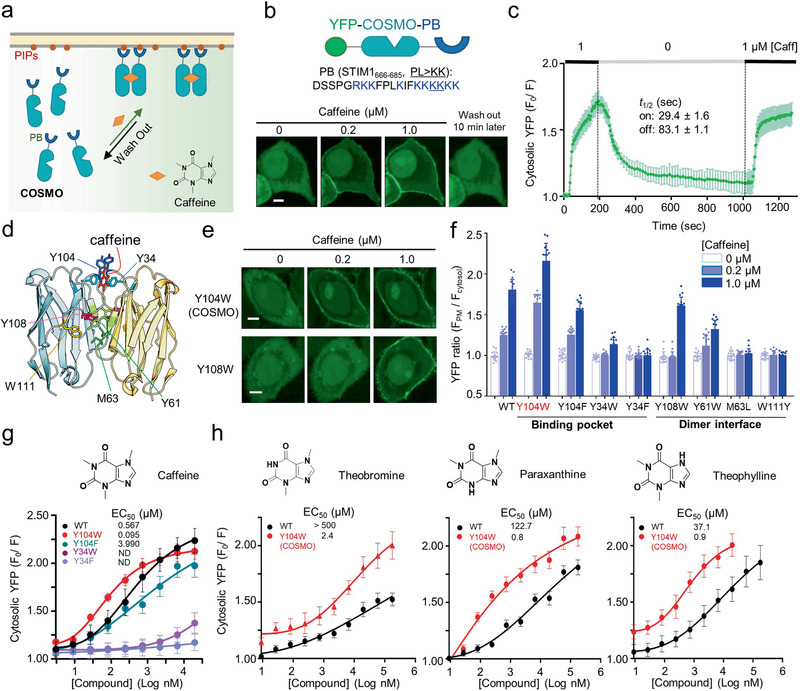
Design of a cytosol‐to‐PM‐translocation assay to screen COSMO in live cells. a) Schematic depicting a cytosol‐to‐plasma membrane (PM) translocation assay. b) High‐content confocal imaging to monitor caffeine‐inducible translocation of YFP–acVHH–PB from the cytosol to PM in HeLa cells. The STIM1–PB sequence information and domain organization of the construct were shown on the top. c) Quantification of the changes in cytosolic YFP–acVHH–PB signals in response to caffeine addition and withdrawal from the culture media. d) The 3D structure of acVHH dimers in complex with caffeine (PDB entry: 6QTL). Key residues nearby the caffeine‐binding pocket and at the dimer interface were indicated. e) Confocal images showing the distribution of YFP–acVHH–PB variants before and after caffeine treatment in HeLa cells. f) Quantification of the PM/cytosol ratio of YFP signals upon addition of 0, 0.2 × 10^−6^, and 1 × 10^−6^ m caffeine to HeLa cells transfected with the indicated constructs. g) Dose–response curves for the indicated acVHH–PB variants upon titration with caffeine. h) Dose‐dependent responses of the indicated variants following treatment with three major caffeine metabolites (theobromine, paraxanthine, and Theophylline). Data were shown as mean ± s.d. Scale bar, 5 µm. *n* = 16 cells from three independent assays.

To facilitate the high‐throughput screening (HTS) of acVHH variants with varying affinities to caffeine, we developed a convenient protein translocation assay by fusing acVHH with a phosphoinositide (PIP)‐binding (PB) domain derived from the stromal interaction molecule 1 (STIM1; residues 666–685; **Figure** **1**a,b; Figure S1, Supporting Information). The PB domain in a dimeric or multimeric form is known to interact with PI(4,5)P_2_ and PI(3,4,5)P_3_ (or PIP3), two phospholipids that are abundantly enriched in the inner half leaflet of the PM.^[^ ^15^ ^]^ Two mutations, P682K/L683K, were introduced to enhance the PIP‐binding capability of the hybrid protein.^[^ ^16^ ^]^ We have previously shown that the cytosol‐to‐PM translocation of the modified PB domain can be initiated by light‐inducible oligomerization.^[^ ^16^ ^]^ In our envisioned design, the acVHH–PB hybrid protein stays in the cytosol in its monomeric form. Upon addition of caffeine, we anticipate to observe its translocation toward PM due to dimerization of PB to increase its avidity toward PM‐resident PIPs (Figure 1a). Indeed, when expressed in HeLa cells, we observed a notable dose‐dependent cytosol‐to‐PM translocation of yellow fluorescent protein (YFP)–acVHH–PB upon addition of caffeine (*t*_1/2, on_ = 29.4 ± 1.6 s; Figure 1b,c). After withdrawal of caffeine, the PM‐bound fraction of acVHH–PB returned to the cytosol with a deactivation half‐life of 83.1 ± 1.1 s (Figure 1b,c; Movie S1, Supporting Information), clearly attesting to the reversibility of this synthetic system.

The PM‐translocation assay enabled us to screen acVHH mutants at real time in living cells to identify potent caffeine binders. Combined with site‐directed mutagenesis, we sought to identify key residues that would enhance the affinity of acVHH to caffeine. We focused on performing mutagenesis on residues located in close proximity to the caffeine‐binding pocket and at the VHH/VHH dimer interface area^[^ ^17^ ^]^ (Figure 1d; Figure S1, Supporting Information). Among all the tested variants, Y104W stood out as the best construct, which displayed the highest degree of PM translocation upon addition of caffeine, and hence renamed as COSMO. Mutations introduced in other positions (e.g., Y34, Y61, M63, Y108, and W111) were found to reduce or even abrogate caffeine‐inducible PM translocation (Figure 1d,f; Figure S2, Supporting Information).

To probe the oligomeric state of WT acVHH and COSMO under physiological conditions in cellulo rather than using artificial recombinant proteins in vitro, we resorted to our previously established mini‐tagging approach by replacing the microtubule binder in MoTag^[^ ^18^ ^]^ with the modified STIM1–PB tag described above (Figure S3, Supporting Information). To establish a calibration curve for assessing protein oligomeric states in live cells, we fused PB domain with well‐known oligomeric proteins (monomeric mCherry, dimeric GST, and tetrameric DsRed) and examined their localization using confocal microscopy (Figure S3b, Supporting Information). We found that the degree of PM translocation showed a positive correlation with the protein oligomeric states (Figure S3, Supporting Information). The use of the PB tag to determine protein oligomeric states was further validated by rapamycin inducible dimerization (FKBP–FRB fusion) and tetramerization (FRB–FKBP fusion) systems^[^ ^19^ ^]^ (Figure S3d,e, Supporting Information). Clearly, the PB‐tagging method can be applied to quantitatively discriminate proteins assembled as monomer, dimer, or tetramer in single cells (Figure S3d, Supporting Information). When the similar method was extended to analyze acVHH and COSMO, we found that both proteins seemed to exist as dimer in the presence of caffeine (Figure 1f; Figure S2c, Supporting Information).

The PM‐translocation assay also provided us a unique opportunity to quantitatively probe the strengths of caffeine binding to the engineered acVHH variants in living cells. To achieve this, we titrated caffeine into HeLa cells expressing YFP–acVHH–PB variants (as exemplified in Figure S2c in the Supporting Information) and used the PM‐to‐cytosol ratio of fluorescent signal as a sensitive readout. WT acVHH showed an apparent EC_50_ value of 567.5 × 10^−9^ m (Figure 1g), which is comparable to the dissociation constant (*K*_d_ = 500 × 10^−9^ m) determined in aqueous solutions using purified protein.^[^ ^9^, ^11^ ^]^ By contrast, COSMO (acVHH–Y104W) showed higher sensitivity to caffeine, with the EC_50_ value being reduced by over sixfold (95.1 × 10^−9^ m; Figure 1g and **Table** **1**; Movie S2, Supporting Information). Moreover, the EC_50_ value of COSMO was largely unaffected by its expression level when assessed in cellulo (Figure S4, Supporting Information). In the 3D structure of a caffeine‐bound dimeric acVHH complex, two Y104 residues from neighboring acVHH molecules are situated right above caffeine to form a “cap” (Figure 1). A water molecule seems to stabilize the cap and prevent the escape of bound caffeine via formation of hydrogen bonds (Figure S2a, Supporting Information). The Y‐to‐W replacement could still preserve the hydrogen bonds considering the H‐bond forming ability of its aromatic (*ϕ*) ring with the OH group of the water^[^ ^20^ ^]^ (Figure S2a, Supporting Information). In support of this view, the Y‐to‐F substitution, which led to a complete loss of H‐bonds in the aromatic cap, resulted in great loss of binding (EC_50_ = 3990.2 × 10^−9^ vs 567.5 × 10^−9^ m; Figure 1g and Table 1). Meanwhile, the *π*–*π* stacking interaction between Y34 and the sandwiched caffeine appeared to be essential for acVHH dimerization as replacement of Y34 with F or W substantially attenuated or even abrogated caffeine‐induced effects (Figure 1g and Table 1; Figure S2b, Supporting Information).

**Table 1:** Summary of caffeine sensitivity (EC_50_ values) for representative acVHH variants and biCOSMO variants tested in the study. *n* = 16 cells from three independent assays. Variants with functional validation were highlighted in bold

Variants^a)^	EC_50_
WT	(567.5 ± 1.6) × 10^−9^ m
**Y104W (COSMO)**	(95.1 ± 1.2) × 10^−9^ m
Y104F	(3990.2 ± 4.7) × 10^−9^ m
Y34W	>20 × 10^−6^ m
Y34F	No binding
Y108W	>2 × 10^−6^ m
Y108F	No binding
Y61W	>1 × 10^−6^ m
Y61F	No binding
F39W	No binding
F39Y	No binding
W111F	No binding
W111Y	No binding
M63L	No binding
**biCOSMO‐S**	(16.9 ± 2.0) × 10^−9^ m
**biCOSMO‐L**	Intramolecular binding
2 × WT acVHH‐S	(530.9 ± 1.6) × 10^−9^ m

Considering that caffeine can be metabolized in the liver of mammals into paraxanthine (84%), theobromine (12%), and theophylline (4%)^[^ ^21^ ^]^ and that WT acVHH shows weak or no appreciable binding to these metabolites,^[^ ^9^, ^22^ ^]^ we next sought to test if COSMO could respond to these chemicals and thus expand its effective substrate scope. As anticipated, COSMO exhibited 41–217‐fold enhancement in the binding strength toward the three major caffeine metabolites when compared to WT acVHH (Figure 1h). However, given the relatively large gap in the sensitivity between caffeine and its metabolites (over 25‐fold difference), caffeine can still achieve specific activation of COSMO when used in the range of (10–100) × 10^−9^ m (Figure 1h; Figure S5, Supporting Information). Under this relatively low dose, COSMO remained largely inert to caffeine metabolites and analogs.

Next, we expanded the scope of COSMO application with commercially available caffeinated beverages consumed in our daily life, including Starbucks coffee, Red Bull, and Coco Cola (Figure S6, Supporting Information). In HeLa cells incubated with diluted beverages, we invariantly observed a dose‐dependent, cytosol‐to‐PM translocation of YFP–COSMO–PB even at a dilution factor of 1:20 000 (Figure S6, Supporting Information). Collectively, through rationalized mutagenesis and screening, we have identified COSMO as a potent genetically encoded synthetic module that can be used to control protein homodimerization using caffeine, caffeine metabolites (also available from chocolate or coco), as well as a broad range of caffeinated beverages consumed in our daily life. This attractive feature makes COSMO a more economic and biocompatible CID system compared to existing chemogenetic tools. Clearly, the engineered COSMO system is fully controllable with the daily consumed amounts of caffeine ((1–10) × 10^−6^ m effective plasma concentration) in the Western society.

### 2.2 Application of COSMO as a Chemically Inducible Dimerizer

**Figure 2:**
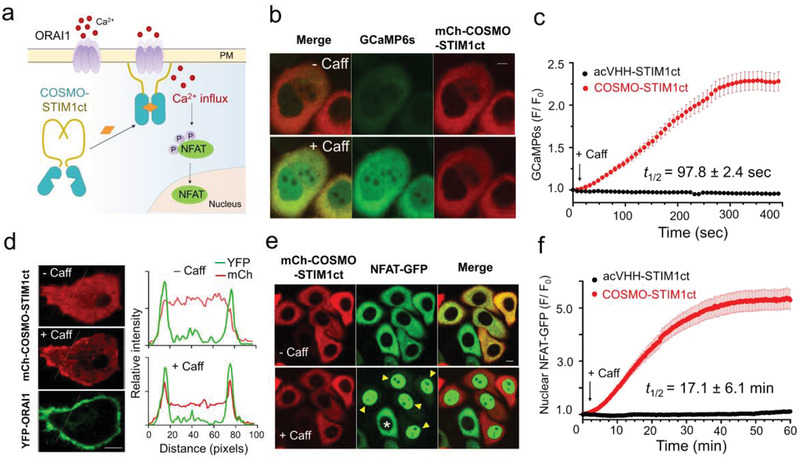
Chemogenetic control of Ca^2+^ entry and nuclear translocation of NFAT in HeLa cells using the COSMO system. a) Schematic illustrating the design of caffeine‐gated Ca^2+^ channels. b) Monitoring Ca^2+^ influx by GCaMP6s fluorescence (green) in HeLa cells co‐expressing mCh–COSMO–STIM1ct (red) before and after 1 × 10^−6^ m caffeine treatment. c) Quantification of cytosolic Ca^2+^ changes following addition of 1 × 10^−6^ m caffeine in HeLa cells co‐expressing the indicated constructs. *n* = 40–60 cells from three independent assays. d) Confocal images showing the localization of mCherry–COSMO–STIM1ct before and after caffeine treatment. HeLa cells were co‐transfected with YFP–ORAI1. The graphs on the right showing the quantification of mCh and YFP signals across the dashed line. e) Confocal images of HeLa cells co‐expressing NFAT–green fluorescent protein (GFP) and mCh–COSMO–STIM1ct before and after 1 × 10^−6^ m caffeine treatment. Arrowheads, mCh‐positive cells with NFAT nuclear entry; asterisk: mCh‐negative cells showing no nuclear translocation of NFAT–GFP. f) Quantification of nuclear accumulation of NFAT–GFP following addition of 1 × 10^−6^ m caffeine in HeLa cells expressing the indicated hybrid constructs. Data were shown as mean ± s.d. Scale bar, 5 µm. *n* = 40–60 cells from three independent assays.

We moved on to test the feasibility of using COSMO in biological context as a chemically inducible dimerizer. We sought to design a synthetic device that can use low‐dose caffeine to switch on theORAIcalcium channel and control the downstream calcium‐responsive transcription factor, the nuclear factor of activated T cells (NFAT).^[^ ^23^ ^]^ Because forced dimerization of the N‐terminus of the cytoplasmic domain of STIM1 (STIM1ct) has been shown to switch on autoinhibitory STIM1 to engage and activate ORAI calcium channels,^[^ ^15^, ^16^, ^24^, ^25^ ^]^ we conjectured that caffeine‐inducible dimerization of STIM1ct could likewise achieve the same function (**Figure** **2**a). We first made the acVHH–STIM1ct hybrid construct but failed to observe pronounced caffeine‐induced calcium influx in HeLa cells incubated with 1 × 10^−6^ m caffeine (Figure S7a, Supporting Information). After replacing acVHH with COSMO, we detected a robust calcium influx with an activation half‐life of 97.8 ± 2.4 s and observed a notable co‐localization of COSMO‐STIM1ct with PM‐embedded ORAI1 channels in the presence of caffeine (Figure 2b–d), indicating the physical coupling between engineered STIM1 and ORAI1.^[^ ^23^ ^]^ Given that sustained Ca^2+^ influx can further activate a Ca^2+^‐dependent phosphatase, calcineurin, and subsequently dephosphorylate the downstream calcium‐responsive NFAT to cause its nuclear translocation,^[^ ^26^ ^]^ we further compared the degree of NFAT nuclear localization before and after caffeine treatment. In agreement with the calcium influx data, caffeine caused efficient shuttling of cytosolic NFAT into the nuclei in all cells expressing COSMO–STIM1ct within 30 min (*t*_1/2_ = 17.1 min; Figure 2e), but not in those transfected with acVHH–STIM1ct (Figure S7b, Supporting Information). Taken together, these data establish COSMO as a more efficient and potent CID system compared to WT acVHH.

**Figure 3:**
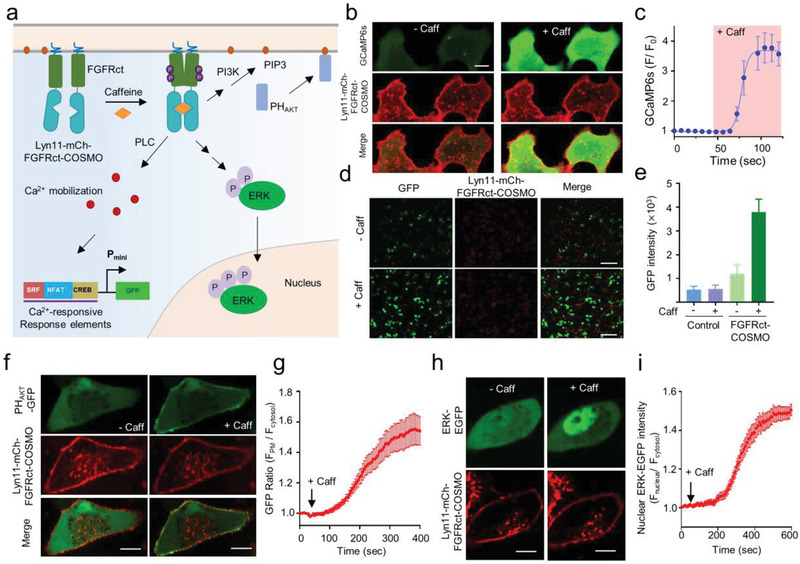
Caffeine‐inducible activation of FGFR‐mediated cell signaling with the COSMO system. Data were shown as mean ± s.e.m. a) Schematic illustrating caffeine‐inducible activation of the PM‐anchored cytoplasmic region of FGFR (FGFRct) and list downstream effectors. b) Monitoring intracellular Ca^2+^ mobilization arising from phosphoinositide phospholipase C‐*γ* (PLC*γ*) activation by GCaMP6s (green) in HeLa cells co‐expressing Lyn11–mCh–FGFRct–COSMO (red) before and after 1 × 10^−6^ m caffeine treatment. Scale bar, 10 µm. c) Quantification of cytosolic Ca^2+^ mobilization by GCaMP6s signals shown in panel (b). *n* = 16 cells from three independent assays. d) Representative confocal images showing the GFP reporter expression before and after 1 × 10^−6^ m caffeine treatment. HEK293T cells were co‐transfected with Lyn11–mCh– FGFRct–COSMO and GFP reporters, the expression of which is driven by synthetic Ca^2+^‐responsive transcriptional response elements derived from serum response factor (SRF), nuclear factor of activated T cells (NFAT), and the cAMP response element‐binding protein (CREB). Scale bar, 100 µm. e) Quantification of GFP reporter expression shown in panel (d). Cells only transfected with the reporter cassette were used as control. *n* = 150 cells from three independent assays. f) Fluorescence images of HeLa cells expressing Lyn11–mCh–FGFRct–COSMO and PH_AKT_–GFP before and after treatment with caffeine. g) Time course showing changes in PH_AKT_–GFP during the cytosol to PM translocation following caffeine‐induced activation of FGFRct. *n* = 16 cells from three independent assays. h) Fluorescence images of HeLa cells expressing Lyn11–mCh–FGFRct–COSMO and ERK–GFP before and after caffeine treatment. Scale bar, 10 µm. i) Quantification of nuclear translocation of ERK–GFP following addition of 1 × 10^−6^ m caffeine. *n* = 16 cells from three independent assays.

Encouraged by the excellent performance of COSMO, we further set out to engineer PM‐resident tyrosine receptor kinases (RTKs), aiming to use caffeine to replace growth factors to recapitulate RTK‐mediated intracellular signaling. To explore this idea, we fused the COSMO module with cytoplasmic region of fibroblast growth factor receptor 1 (FGFRct) and tethered the hybrid protein toward PM via N‐terminal tagging with the Lyn11 motif (**Figure** **3**a). We envisioned that dimerization of FGFRct induced by caffeine could bring two FGFRct molecules into close proximity and subsequently activate this receptor as its natural ligand FGF does.^[^ ^27^ ^]^ To monitor FGFR signaling at real time in living cells, we focused on assessing three hallmark downstream signals (Figure 3a): intracellular Ca^2+^ rise due to phospholipase C activation (GCaMP6s as reporter), PIP3 synthesis in the PM because of phosphoinositide 3‐kinaes (PI3K) activation (PH_AKT_–GFP as a biosensor for PIP_3_), and the nuclear translocation of the extracellular signal‐regulated kinase (ERK). Following the addition of caffeine, we observed a robust increase in intracellular Ca^2+^ as reflected by the over threefold enhancement of GCaMP6s fluorescence within seconds in HeLa cells expressing Lyn11–mCh–FGFRct–COSMO (Figure 3b,c). Caffeine‐triggered calcium mobilization could be further employed to drive the expression of genes of interest (e.g., GFP as a reporter) by taking advantage of synthetic Ca^2+^‐sensitive transcriptional response elements derived from NFAT, serum response factor (SRF), and cyclic adenosine monophosphate (cAMP) response element‐binding protein (CREB) (Figure 3d,e). Meanwhile, time‐lapse live cell imaging revealed that two additional downstream effectors, PI3K and ERK, were both activated within 5–10 min, which were reported by the cytosol‐to‐PM translocation of a PIP3 sensor PH_AKT_–GFP (Figure 3f,g) and the nuclear entry of ERK–GFP (Figure 3h,i). Collectively, these findings establish the compatibility of COSMO with membrane‐bound signaling receptors to rewire RTK‐mediated cell signaling with caffeine, thus obviating the use of pleotropic growth factors that tend to cause crosstalks among various RTKs to confer better signaling fidelity.

**Figure 4:**
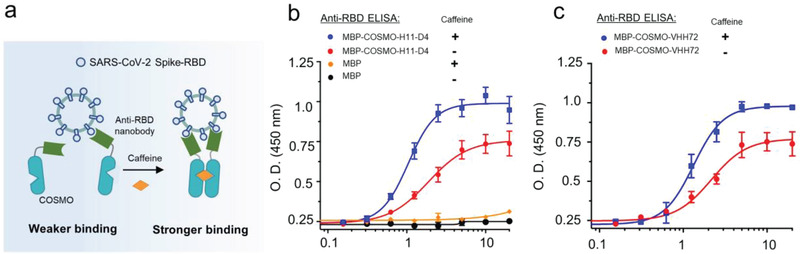
Use of COSMO to enhance the binding of nanobodies against the RBD domain from the SARS‐CoV‐2 spike protein. a) Schematic illustration of caffeine‐inducible COSMO dimerization to form a noncovalently connected bivalent nanobody, thereby mimicking the role of Fc homodimerization module and enhancing its antigen recognition capability to engage the viral target. b,c) ELISA assessment of the reactivity of two COSMO‐tagged anti‐RBD nanobodies, b) H11‐D4 and c) VHH72, against SARS‐CoV‐2 RBD. Maltose binding protein (MBP) was used as a control. Datapoints represent the mean of three replicates and data were shown as mean ± s.e.m.

Next, we speculated that COSMO can be modularly tagged to nanobodies that lack the bulky Fc fragment of a typical antibody, and enable the assembly of noncovalent bivalent nanobodies to enhance their antigen recognition. To explore this exciting possibility, we fused COSMO with two newly developed nanobodies (VHH72^[^ ^28^ ^]^ and H11‐D4^[^ ^29^ ^]^) that specifically recognize the receptor‐binding domain (RBD) of the spike protein derived from the severe acute respiratory distress (SARS) coronavirus 2 (SARS‐CoV‐2). These nanobodies have the potential to mitigate SARS‐CoV‐2 infection and alleviate COVID‐19 symptoms by blocking the interaction of viral spike protein with the angiotensin‐converting enzyme 2 (ACE2) expressed on the surface of human lung epithelial cells.^[^ ^30^ ^]^ Because the use of a covalently linked bivalent nanobody or fusion with the Fc homodimerization fragment could enhance the nanobody–target interaction and virus neutralization capability,^[^ ^28^, ^29^ ^]^ we asked whether chemical‐inducible dimerization of these nanobodies could exhibit similar boosting effects (**Figure** **4**a). After screening several anti‐Spike nanobodies fused with COSMO, we found that COSMO–H11‐D4 and COSMO–VHH72 showed stronger binding to SARS‐CoV‐2 RBD in the presence of caffeine based on enzyme‐linked immunosorbent assay (ELISA) results (Figure 4b,c; Figure S8, Supporting Information). Their apparently binding affinities were improved by 60–80% (COSMO–H11‐D4: 25 × 10^−9^ vs 14 × 10^−9^ m; COSMO–VHH72: 29 × 10^−9^ vs 18 × 10^−9^ m). Evidently, chemically induced dimerization could be exploited to enhance the binding strength of nanobodies against the SARS‐CoV‐2 spike protein, which promises to more effectively neutralize the virus infectivity. We anticipate that the COSMO system could offer a modular strategy to conditionally tune the activity of nanobodies.

### 2.3 COSMO Concatemer (biCOSMO) as a Modular Switch to Control Protein Activities

**Figure 5:**
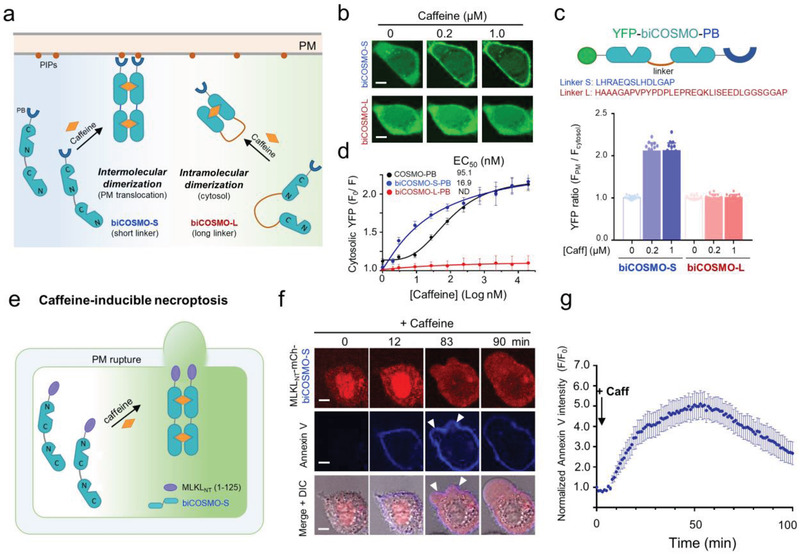
biCOSMO‐S as a potent chemical dimerizer to control necroptosis of HeLa cervical cancer cells. Scale bar, 5 µm. a) Schematic illustration of two possible caffeine‐induced effects on a dimeric COSMO concatemer (biCOSMO). The N‐ and C‐termini of two consecutive COSMO proteins are separated by a distance of ≈45 Å. A short linker (biCOSMO‐S) with an estimated length of <45 Å favors intermolecular dimerization over intramolecular dimerization. This short rigid linker is derived from the initial segment of the coiled‐coil region 1 (CC1) of STIM1. b) Confocal images showing the subcellular localization of the indicated YFP–biCOSMO–PB constructs (green) in HeLa cells before and after 1 × 10^−6^ m caffeine treatment. c) Quantification of the PM/cytosol ratio of YFP signals upon addition of 0, 0.2 × 10^−6^, and 1 × 10^−6^ m caffeine to HeLa cells transfected with the indicated biCOSMO variants. The linker sequences were shown above the bar graphs. Data were shown as mean ± s.d. *n* = 16 cells from three independent assays. d) Dose–response curves for the indicated biCOSMO–PB constructs. Data were shown as mean ± s.d. *n* = 16 cells from three independent assays. e) Design of a synthetic cancer cell suicide device using biCOSMO‐S. f) Time‐lapsed imaging of HeLa cells expressing MLKL_NT_–mCh–biCOSMO–S (red) upon addition of 1 × 10^−6^ m caffeine. Annexin V conjugated with Pacific Blue (blue) was used to stain dead cells. DIC, differential interference contrast. g) The time course of caffeine‐induced necroptotic cell death reported by cell surface staining with Annexin V. Data were shown as mean ± s.e.m. *n* = 10 cells from three independent assays. The second phase of intensity decline after 50 min was due to PM rupture and cell death.

Although COSMO exhibited less EC_50_ to caffeine (95.1 × 10^−9^ m) and greatly expanded its substrates, it appears to still fall behind the performance of the FKBP–FRB–rapamycin system (*K*_d_ = (10–20) × 10^−9^ m). To generate a more potent COSMO‐based system, we attempted to assemble COSMO in a concatemeric form with linkers of different lengths (**Figure** **5**a). When we screened these 2×COSMO–PB (biCOSMO–PB) constructs with 0.2 × 10^−6^ and 1 × 10^−6^ m caffeine as described above, we observed that bivalent acVHH variants covalently connected with short flexible linkers (L1–L3) showed stronger cytosol‐to‐PM translocation (Figure S9, Supporting Information). However, the same linkers grafted into COSMO led to predimerization of biCOSMO–PB, as reflected by noticeable PM decoration in the absence of caffeine. We speculated that limiting the flexibility of bivalent COSMO using a more rigid linker might mitigate this issue. Therefore, we resorted to an 11‐mer sequence derived from the coiled‐coil 1 (CC1) domain of STIM1 (residues L251–L261), which has a strong helical propensity and adopts a helical structure (Figure S9, Supporting Information). Indeed, this new design (named biCOSMO‐S) substantially reduced the basal activity, as judged from the lack of discernible PM translocation of biCOSMO–PB (Figure 5b,c). More importantly, the caffeine sensitivity was improved by over 30‐fold compared to WT acVHH (EC_50_ = 16.9 × 10^−9^ vs 567.5 × 10^−9^ m; Figure 5d and Table 1; Movie S3, Supporting Information). However, when we constructed a similar concatemer using WT acVHH with the same linker (2×acVHH‐S–PB), we only observed slight improvement in the performance compared to acVHH–PB (530.9 × 10^−9^ vs 567.5 × 10^−9^ m, Table 1), which highlights the uniqueness of the COSMO module to enhance the binding affinity. Surprisingly, when we used a longer linker with a mixed flexible and rigid structure to connect two COSMO molecules (biCOSMO‐L–PB), we noted that caffeine‐induced PM translocation was no longer observed (Figure 5b–d; Movie S3, Supporting Information). We speculated that biCOSMO‐L–PB might favor an intramolecular dimerization (as seen in UniRapR14) instead of intermolecular dimerization as seen with biCOSMO‐S (Figure 5a).

To further validate that biCOSMO‐S could be harnessed to induce intermolecular dimerization, we set out to devise a chemically inducible cell suicide device by fusing biCOSMO‐S with the N‐terminal domain of mixed lineage kinase domain like pseudokinase (MLKL_NT_). Once activated via phosphorylation upon inflammatory stimulation, the N‐terminal region of MLKL is able to oligomerize and translocate toward the PM to perforate membrane and cause necroptosis, a new form of nonapoptotic cell death.^[^ ^31^ ^]^ We therefore speculated that caffeine‐inducible dimerization of MLKL_NT_–biCOSMO‐S could similarly trigger necroptosis but obviate the need of detrimental necroptotic stimuli (Figure 5e). Upon caffeine treatment, we observed a notable translocation of MLKL_NT_–mCh–biCOSMO‐S from the cytosol toward the PM, accompanied with positive staining for Annexin V (cell death indicator) and ultimate necroptotic bubble formation and PM rupture (Figure 5f,g; Movie S4, Supporting Information). Collectively, these findings establish biCOSMO‐S as a potent chemically inducible dimerizer that can be engineered to kill cancer cells within hours, which will likely find use as a safety switch during adoptive cell therapies.

**Figure 6:**
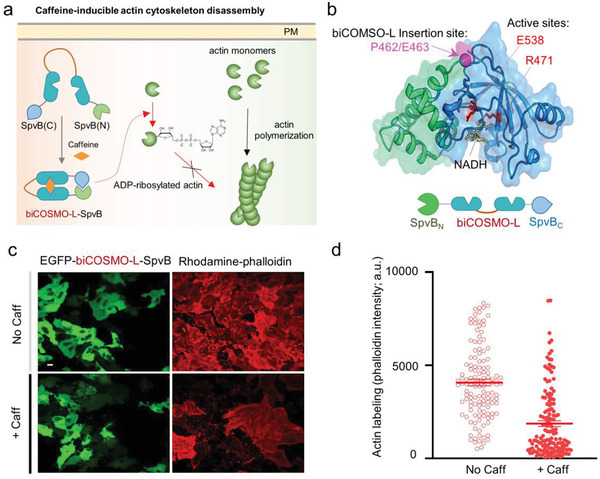
Design of a caffeine‐switchable, genetically encoded inhibitor of actin cytoskeleton assembly based on biCOSMO‐L. a) Schematic illustration of caffeine‐inducible actin cytoskeleton disassembly. biCOSMO‐L is inserted between SpvB (N) (aa 375–462) and SpvB (C) (aa 463–591). b) The 3D structure of an ADP‐ribosyltransferase (ATR) domain derived from *Salmonella* SpvB (PDB entry: 3GWL). The domain architecture of the construct was shown below the cartoon. Green, SpvB_ATR N‐domain; blue, SpvB_ATR C‐domain; magenta, biCOSMO‐L insertion site between P461 and E462; red, active sites that catalyze actin ADP ribosylation using nicotinamide adenine dinucleotide (NAD^+^) as substrate. c) Confocal images showing EGFP–biCOSMO‐L–SpvB‐expressing HeLa cells (green) stained with rhodamine‐conjugated phalloidin (red) before and after caffeine treatment. d) Quantification of phalloidin staining results before and after caffeine treatment (as shown in panel (c)). *n* = 130–150 cells from three independent assays.

Finally, we asked whether biCOSMO‐L could be used as a modular allosteric switch to control enzymatic activity. We tested our idea by engineering the adenosine diphosphate (ADP) ‐ribosyltransferase (ATR) domain derived from *Salmonella* SpvB, a bacterial enzyme that catalyzes ADP ribosylation of actin to prevent actin polymerization (**Figure** **6**a).^[^ ^32^, ^33^ ^]^ We inserted biCOSMO‐L into a flexible loop region connecting the N‐ and C‐domains of SpvB (Figure 6b), anticipating that the addition of caffeine would induce the reassembly of a functional SpvB to restore its function to disrupt actin cytoskeleton (Figure 6a). In HeLa cells transfected with enhanced green fluorescent protein (EGFP)–biCOSMO‐L–SpvB, we found that both EGFP‐negative and EGFP‐positive cells showed strong staining of actin polymers by fluorophore‐conjugated phalloidin in the absence of caffeine (Figure 6c). However, upon incubation with caffeine, we observed a substantial reduction or disappearance of actin labeling by rhodamine–phalloidin staining in EGFP‐positive cells (Figure 6c,d), suggesting that caffeine restored the SpvB enzymatic activity to antagonize actin polymerization. This effect was very specific since EGFP‐negative cells without expression of the hybrid construct in the same imaging field still retained strong actin labeling (Figure 6c). In aggregate, we have demonstrated the use of biCOSMO‐L as a caffeine‐controllable switch to control the disassembly actin cytoskeleton. Our engineering strategy will likely open up many more opportunities to remotely interrogate cellular processes via a simple insertion of biCOSMO‐L in signaling proteins or enzymes.

## 3 Discussion

In summary, the COSMO and biCOSMO modules described in the study provide a previously unidentified platform for chemogenetic manipulation of biological processes with caffeine, its metabolites, or even caffeine‐containing beverages. As caffeine is cheap and readily available in daily drinks, these chemical biology tools will greatly reduce the cost in engineering proteins or cells of therapeutic potentials. In theory, the amount of caffeine from a single cup of Starbucks coffee (100–200 mg in 350 mL; reaching an approximate peak plasma concentration of 2.5–5 mg L^−1^ or (13–26) × 10^−6^ m in a 70 kg adult) or a can of soda (46 mg in a 355 mL Coke) is sufficient to activate COSMO or biCOSMO, but far below the amount of caffeine addition or intoxication (75–100 cups of coffee for an adult; or over 1 g) to cause severe insomnia or dizziness due to awakening and cardiotonic actions. Although caffeine is known to enhance calcium‐induced calcium release through the ryanodine receptor, it requires high micromolar to low millimolar range of caffeine, which is at least 1000‐fold higher than the EC_50_ of biCOSMO.^[^ ^34^ ^]^ Hence, the side effects associated with low‐dose caffeine or its metabolites’ usage in the COSMO system are likely to be minimal. These desirable features pave the way for future in vivo application of the COSMO‐based CID system.

Compared to other commonly used CID systems (Table S1, Supporting Information), desirable features of the COSMO platform include i) the least chemical complexity and smallest size of the ligand (194 Da; Figure S10, Supporting Information), ii) a single‐component system with a compact size (only 118 residues for COSMO), iii) low cost and easy accessibility from daily‐consumed beverages and food, iv) excellent bioavailability and compatibility with multiple routes of administration, and v) good reversibility upon metabolism or withdrawal of ligand treatment. Moreover, by inserting a short rigid linker between two copies of COSMO, we have further enhanced the EC_50_ to the low nanomolar range (comparable to the widely used FKBP/FRB/rapamycin system), thereby providing an alternative option for chemogenetic applications. Most importantly, biCOSMO‐L can be modularly inserted into a host protein to enable allosteric control of protein activities and achieve tailored function. We anticipate that COSMO‐derived tools will likely find broad applications in the precise control of therapeutic cells and biologics using caffeine or caffeinated beverages in the near future.

## 4 Experimental Section

##### Molecular Cloning and Plasmids Construction

Plasmids construction was performed using the standard restriction enzyme digestion and ligation method. KOD Hot Start DNA polymerase was purchased from EMD Millipore (Burlington, MA, USA) and used for polymerase chain reaction (PCR) amplifications. Oligonucleotides were synthesized by Sigma–Aldrich (St. Louis, MO, USA). The T4 DNA ligase kit and NEBuilder HiFi DNA Assembly Master Mix were purchased from New England BioLabs (Ipswich, MA, USA). QuikChange Multi Site‐Directed Mutagenesis Kit was obtained from Agilent Technologies (Santa Clara, CA, USA). Recombinant SARS‐CoV‐2 Spike RBD in a biotinylated form was purchased from R&D Systems (Cat No. BT10500; Minneapolis, MN, USA).

YFP–acVHH–PB was generated by inserting the synthesized complementary DNA (cDNA) encoding acVHH (GENEWIT, South Plainfield, NJ, USA) upstream of the STIM1–PB domain into the pEYFP‐C1 vector. To produce oligomeric proteins fused to PB, acVHH or YFP was replaced by mCherry, GST, DsRed, and the FRB/FKBP system, respectively. YFP–acVHH–PB variants were made by using the QuikChange Site‐Directed Mutagenesis Kit. YFP–biCOSMO‐S–PB and YFP–biCOSMO‐L–PB were constructed by inserting amplified acVHH–Y104W with the corresponding linker into YFP–COSMO–PB. mCherry–acVHH (WT/Y104W)–STIM1ct constructs were prepared by amplifying the STIM1ct fragment (233–685) and acVHH (WT/Y104W) via standard PCR and then inserted into a modified pmCherry‐C1 vector. Lyn11–mCh–FGFRct–COSMO was constructed by amplifying cDNAs encoding Lyn11, FGFRct, and acVHH–Y104W, followed by insertion into a pTriEx vector. Bacterial expression vectors encoding maltose binding protein (MBP)–COSMO–H11–D4 and MBP–COSMO–VHH72 were made by amplifying acVHH–Y104W and synthetic cDNAs encoding H11‐D4 or VHH72 and inserting them into the pMCSG‐9 vector, respectively. MLKL_NT_–mCh–biCOSMO‐S was constructed by inserting amplified biCOSMO‐S into the pLentiBlast vector. biCOSMO‐L‐SpvB was constructed by sequentially inserting amplified SpvB N‐ (residues 375–462), biCOSMO‐L and SpvB C‐domain (residues 463–591) into the pEGFP‐C1 vector.

##### Chemical Reagents and Caffeinated Beverages

Caffeine, paraxanthine, theobromine, rapamycin, isopropyl‐*b*‐d‐thiogalactopyranoside (IPTG), dimethyl sulfoxide (DMSO), and theophylline were purchased from Sigma. DMSO stock solution was made refresh prior to use. Annexin V (Pacific Blue conjugate) was purchased from Fisher Scientific. Caffeinated beverages, including Coca Cola, Starbucks coffee, and Red Bull, were purchased from a local grocery store.

##### Cell Culture and Transfection

The HeLa cell line was purchased from ATCC. Cells were cultured at 37 °C with 5% CO_2_ in Dulbecco's Modified Eagle medium (DMEM; Sigma–Aldrich; St. Louis, MO, USA) supplemented with 10% fetal bovine serum (FBS) and 1% penicillin/streptomycin cocktail. For fluorescence imaging experiments, cells were seeded in 35 mm glass‐bottom dishes (Cellvis, Mountain View, CA, USA). On day 2, transfection was performed when cells reached about 50–70% confluency using the Lipofectamine 3000 (Life Technologies; Carlsbad, CA, USA) reagent by following the manufacturer's instructions. 6 h post‐transfection, cells were replenished with normal DMEM. On days 3–4, transfected cells were mounted on a Nikon confocal microscope stage for imaging.

##### HTS of acVHH Variants

Randomized mutagenesis was performed in key sties involved in caffeine biding and the formation of dimeric interface by using acVHH as the template (a total of 27 sites × 20 = 540 constructs; Figure S1, Supporting Information). The PM translocation of acVHH–PB variants was used as a readout for high‐content imaging. HeLa cells were seeded in a glass‐bottom 96‐well microplate (Cellvis, Mountain View, CA, USA) at a density of 1 × 10^4^ cells per well and cultured in 40 µL DMEM supplemented with 10% FBS in 5% CO_2_ at 37 °C. 12 h later, the constructed plasmids were transfected into HeLa cells with Lipofectamine 3000. After 18 h, the microplate was mounted on the IN Cell Analyzer 6000 (GE) high‐content imaging instrument in the absence of caffeine and fast imaging was performed with four captured views per well. Next, the microplate was incubated with caffeine (1 × 10^−6^ m) for 10 min and re‐imaged using the same parameters. The exported florescent images were then analyzed with an Image J PathFinder plugin for automated membrane detection and quantification.^[^ ^35^ ^]^ The screened top candidate mutations were further characterized by titration with increasing doses of caffeine to obtain the EC_50_ cures in living cells. The PM/cytosol ratios were quantified and plotted against the doses to obtain the apparent binding affinity.

##### Purification of Recombinant Proteins from *Escherichia coli*

*Escherichia coli* strain BL21(DE3) cells (EMD Millipore) were transformed with plasmids encoding MBP–COSMO–VHH72 and MBP–COSMO–H11‐D4, and grown at 37 °C in Luria‐Bertani (LB) medium. Protein expression was induced by the addition of 0.5 × 10^−3^ m IPTG when OD600 was around 0.6–0.8, and incubated at 16 °C for another 16 h. Harvested cells were resuspended in a resuspension buffer containing 20 × 10^−3^ m Tris‐HCl pH 7.4, 137 × 10^−3^ m NaCl, and then sonicated. The cellular debris was removed by centrifugation. The MBP‐fusion protein was enriched by passing the clarified supernatants through amylose resin and further purified via size exclusion chromatography (GE Healthcare).

##### Live‐Cell Imaging and Image Analysis

Fluorescence imaging was performed on a Nikon Eclipse Ti‐E microscope equipped with an A1R‐A1 confocal module with LU‐N4 laser sources (argon ion: 405 and 488 nm; diode: 561 nm) and a live‐cell culture cage (maintaining the temperate at 37 °C with 5% CO_2_). 60× oil or 40× oil lens was used for confocal imaging. The EC_50_ values of acVHH and COSMO variants were determined by incubating HeLa cells with DMEM media containing various concentrations of caffeine, its metabolites, or caffeinated beverages for 2 min. To calculate the changes in cytosolic YFP signals (in the form of *F*/*F*_0_), the “Intensity Line Profile” function in the Nikon Elements software was employed. The titration curves were fitted using a dose–response curve function ([Agonist] vs response—variable slope (four parameters)) using the Prism7 software. A total of 16 cells were selected for each titration curve. All experiments were independently repeated three times.

For measurement of Ca^2+^ influx in HeLa cells co‐expressing the green calcium indicator GCaMP6s and mCherry–acVHH/COSMO–STIM1ct, 488 and 561 nm laser sources were used to excite GFP and mCherry, respectively, at an interval of 8 s. The collected images were analyzed by the NIS‐Elements AR microscope imaging software (Nikon, NIS‐element AR version 4.0). Around 40–60 cells were selected to define regions of interest (ROI) for analyzing the GCaMP6s fluorescence intensity. All experiments were repeated three times.

To monitor NFAT–GFP nuclear translocation, a HeLa cell line was used stably expressing NFAT1_1‐460_–GFP. mCherry–acVHH/COSMO–STIM1ct were transfected into this cell line, which was imaged 24 h after transfection. A total of 60 min time‐lapse imaging was recorded at an interval of 15 s, and the nuclear GFP signal ratio changes (in the format of *F*/*F*_0_) were used to report the efficiency of NFAT activation. At least 40 cells were analyzed for each condition in three independent experiments.

To monitor the necroptosis of HeLa cells transfected with MLKL_NT_–mCherry–biCOSMO‐S, 405 and 561 nm laser sources were used to excite Pacific‐Blue‐conjugated Annexin V and mCherry, respectively. HeLa cells were preincubated with the Annexin V staining reagent for 5 min before imaging, and 100 × 10^−6^ m caffeine was added to the medium to reach a final concentration of 1 × 10^−6^ m during the imaging process. Time‐lapse imaging was carried out at an interval of 1 min for up to 100 min and the blue signal ratio change of Annexin V (*F*/*F*_0_) was used to quantitatively report the necroptotic progression.

##### ELISA to Probe the Nanobody–RBD Interaction

Wells of microtiter plates were coated overnight at 30 °C with 30 ng streptavidin. Biotinylated SARS‐CoV–RBD was added and incubated at 4 °C for 4 h. The coated plates were then blocked with 5% phosphate buffered saline (BSA) in phosphate buffered saline (PBS). Serially diluted nanobodies (20, 10, 5, 2.5, 1.25, 0.63, 0.31, 0.06, and 0.006 µg mL^−1^) were added to the individual wells. Binding was detected by incubating the plates sequentially with a mouse anti‐MBP monoclonal antibody (E8032S, New England Biolabs) and a horseradish peroxidase (HRP)‐linked antimouse immunoglobulin G (IgG) (1/2000, NXA931, GE Healthcare). After washing, 30 µL of tetramethylbenzidine substrate (BD OptETA) was added to each well and the reaction was stopped by addition of 20 µL of 1 m H_2_SO_4_. The absorbance (O.D.) at 450 nm was measured with an iMark Microplate Absorbance Reader (Bio‐Rad). The obtained curves were fitted by using a nonlinear regression binding model (Graphpad 7.0).

##### Statistical Analysis

Quantitative data were shown as mean ± standard error of measurement (s.e.m.) or mean ± standard deviation (s.d.) unless otherwise explained. The analyzed number (*n*) of samples was listed for each experiment. Acquired data were analyzed by Graphpad Prism 7 and Microsoft Excel 2013.

## Conflict of Interest

The authors declare no conflict of interest.

## Supporting information

Supporting Information

Supplemental Video 1

Supplemental Video 2

Supplemental Video 3

Supplemental Video 4
