# Supplementary material for: Caffeine‐Operated Synthetic Modules for Chemogenetic Control of Protein Activities by Life Style
Source: Adv Sci (Weinh). 2020 Dec 13;8(3):2002148. doi: 10.1002/advs.202002148 (PMC7856909; doi:10.1002/advs.202002148)
Supplement: Supplementary file 1 — Supporting Information [file ADVS-8-2002148-s001.pdf]

© 2020 Wiley-VCH GmbH

ADVANCED SCIENCE
Open Access

# Supporting Information

for *Adv. Sci.*, DOI: 10.1002/advs.202002148

# Caffeine-operated synthetic modules for chemogenetic control of protein activities by life style

*Tianlu Wang, Lian He, Ji Jing, Tien-Hung Lan, Tingting Hong, Fen Wang, Yun Huang*, Guolin Ma*, and Yubin Zhou**

# Supporting Information

**Caffeine-operated synthetic modules for chemogenetic control of protein activities by life style**

*Tianlu Wang[+], Lian He[+], Ji Jing, Tien-Hung Lan, Tingting Hong, Fen Wang, Yun Huang*, Guolin Ma*, and Yubin Zhou**

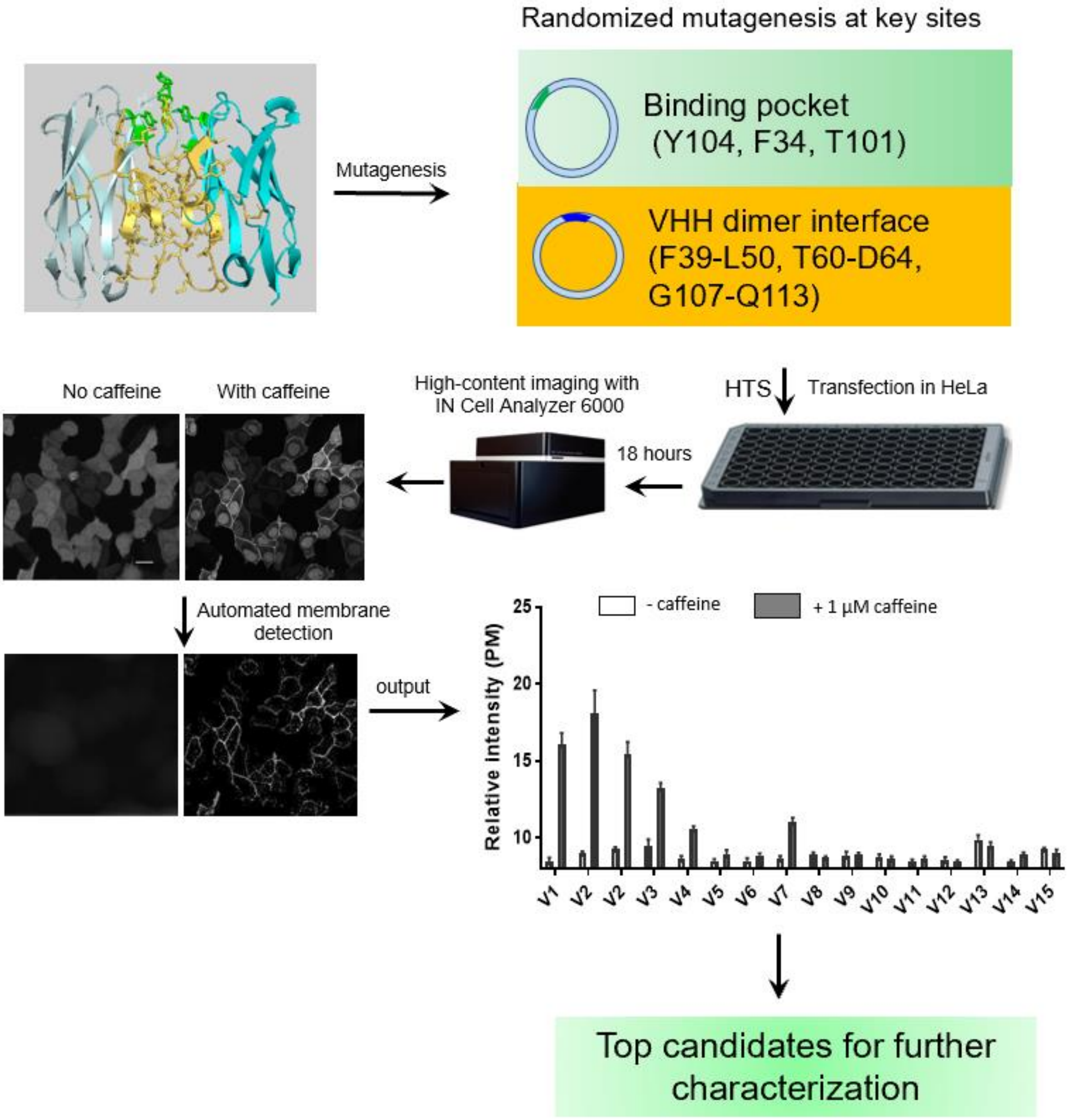

**Figure S1.** A workflow illustrating the screening process of acVHH variants and optimization of COSMO. Mutations were first randomly introduced into key positions/interfaces involved in caffeine binding and dimerization (a total of 27 positions). Each variant was tagged with the PB domain, thus enabling the use of cytosol-to-PM translocation as a convenient readout for the assay. An automated PM detection pipeline was used to aid in the image analysis. The top candidates emerged from the screen were further characterized via caffeine titration to obtain their $EC_{50}$ values.

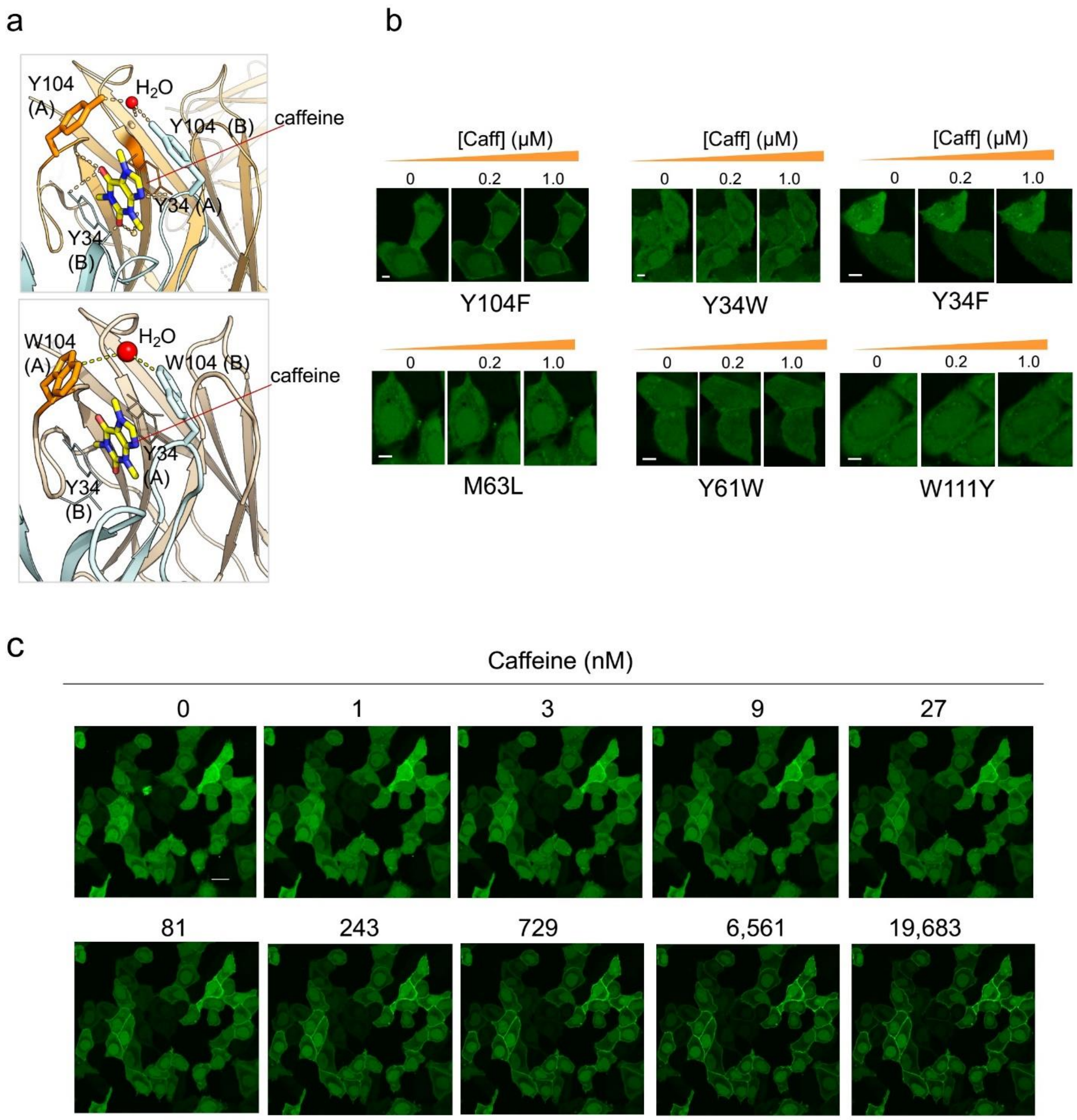


**Figure S2.** Optimization of acVHH variants for chemogenetic control. Scale bar, 5 μm.

a) 3D cartoon representation of the caffeine binding pocket within WT acVHH (PDB entry: 6QT1) and the modeled acVHH-Y104W mutant (COSMO). acVHH dimers were shown in wheat (A) and cyan (B), respectively. Top, Two Y104 residues from the dimeric acVHH sit above caffeine and form hydrogen bonds with $H_2O$ (red). Caffeine (yellow sticks) is sandwiched between two Y34 residues (shown as lines). Bottom, two neighboring W104 residues situate above caffeine and form two putative OH·ϕ H-bonds. b) Confocal images showing the subcellular distribution of YFP-acVHH-PB variants (Y104F, Y34W, Y34F, Y61W, M63L, W111Y) before and after caffeine treatment (0.2 and 1 μM) in HeLa cells. c) Time lapse confocal imaging of YFP-acVHH-PB in HeLa cells titrated with increasing amounts of caffeine (indicated above each image).

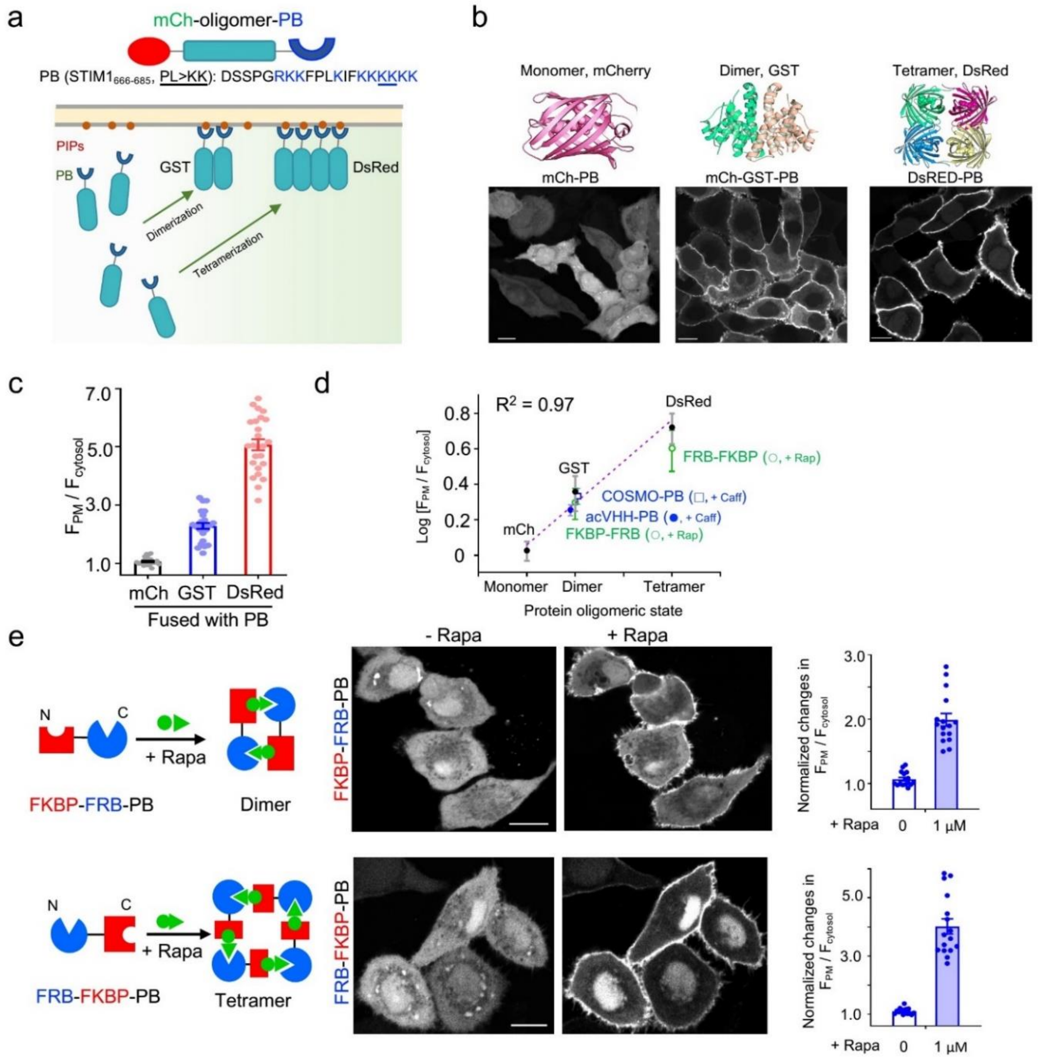


**Figure S3**. Genetically encoded PB-tag for real time dissection of protein assembly in living Cells. Scale bar, 10 µm.

a) Schematic illustrating the design of a modified STIM1 PB domain ($STIM1_{666-685}$; PL>KK mutations) used to probe protein oligomeric states in living cells. A positive correlation between the PM-binding degree of PB domain and the oligomeric state of the fusion partner is anticipated. b) Confocal images of HeLa cells expressing mCherry (mCh)-PB (as monomer) or the indicated oligomeric proteins (GST as dimer and DsRed as tetramer) fused with the PB tag. The 3D structures of mCherry, GST and DsRed were shown on the top. c) Quantification of the PM-to-cytosol ($F_{PM}$/ $F_{cytosol}$) fluorescence intensities in HeLa cells expressing monomeric or oligomeric PB domain (as in panel b). n= 25 cells from 3 independent experiments (mean ± s.e.m.). d) The PM-to-cytosol ratio of fluorescent signals (in Log10 scale) plotted against the oligomeric states of indicated proteins (as shown in panels. c-d). A positive correlation was noted between the two variables ($R^2$ = 0.97). The *in-cellulo* oligomeric state of WT acVHH-PB or COSMO(Y104W)-PB was determined to be comparable with a dimer. e) Rapamycin (Rapa)-inducible FRB/FKBP oligomerization reported by the PB-tag. Left, schematic showing the use of rapamycin to induce FKBP-FRB dimerization (top) or FRB-FKBP tetramerization (bottom). Middle, The representative confocal images of transfected HeLa cells before and after treatment with 1 µM rapamycin. Right, Quantification of the PM-to-cytosol ratio of fluorescent signals from the corresponding images. n = 16 cells from three independent experiments. Data were shown as mean ± s.e.m..

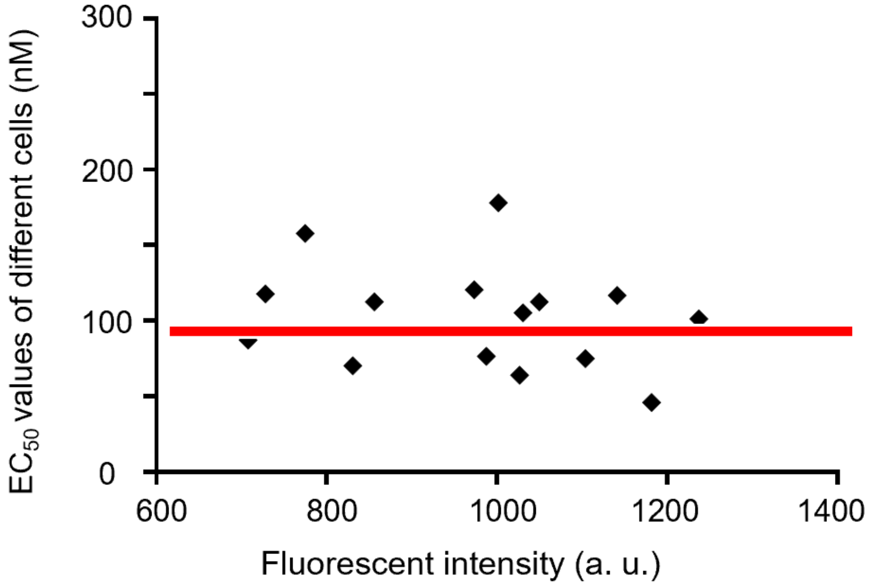


**Figure S4.** $EC_{50}$ values obtained from 15 individual cells expressing various levels of COSMO (indicated by fluorescence intensity of the YFP tag). The red line indicates the averaged $EC_{50}$ value of COSMO for caffeine (95.1 nM). No significant concentration-dependent effect was observed.

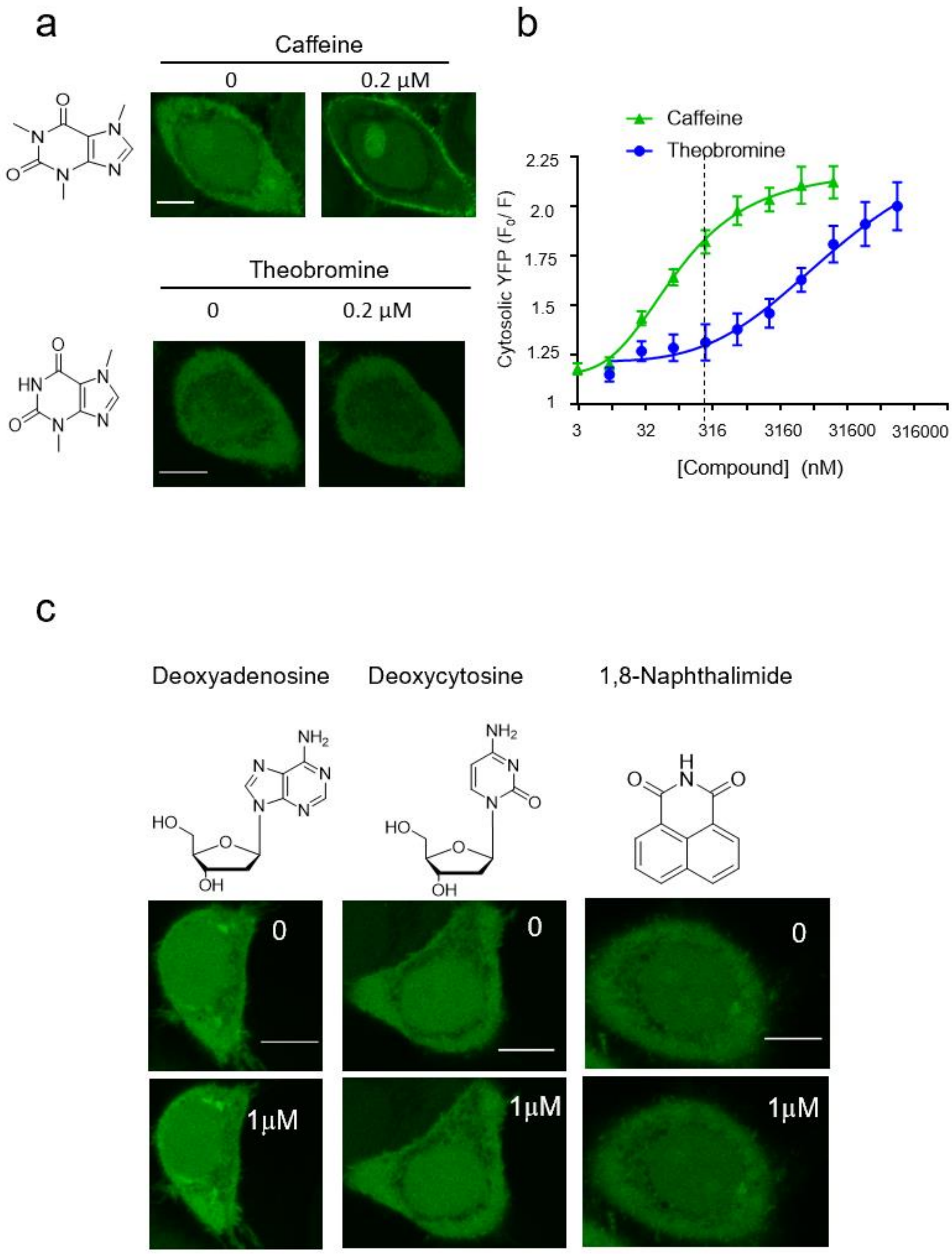


**Figure S5.** Evaluation of the substrate specificity of COMSO. Scale bar, 10 µm. a) Confocal images showing the subcellular distribution of YFP-COSMO-PB in HeLa cells, before and after treatment with caffeine or its major metabolite, theobromine. b) Dose responses curves for COSMO upon titration with caffeine or theobromine. c) Confocal images showing the subcellular distribution of YFP-COSMO-PB before and after treatment with caffeine analogues (1 µM).

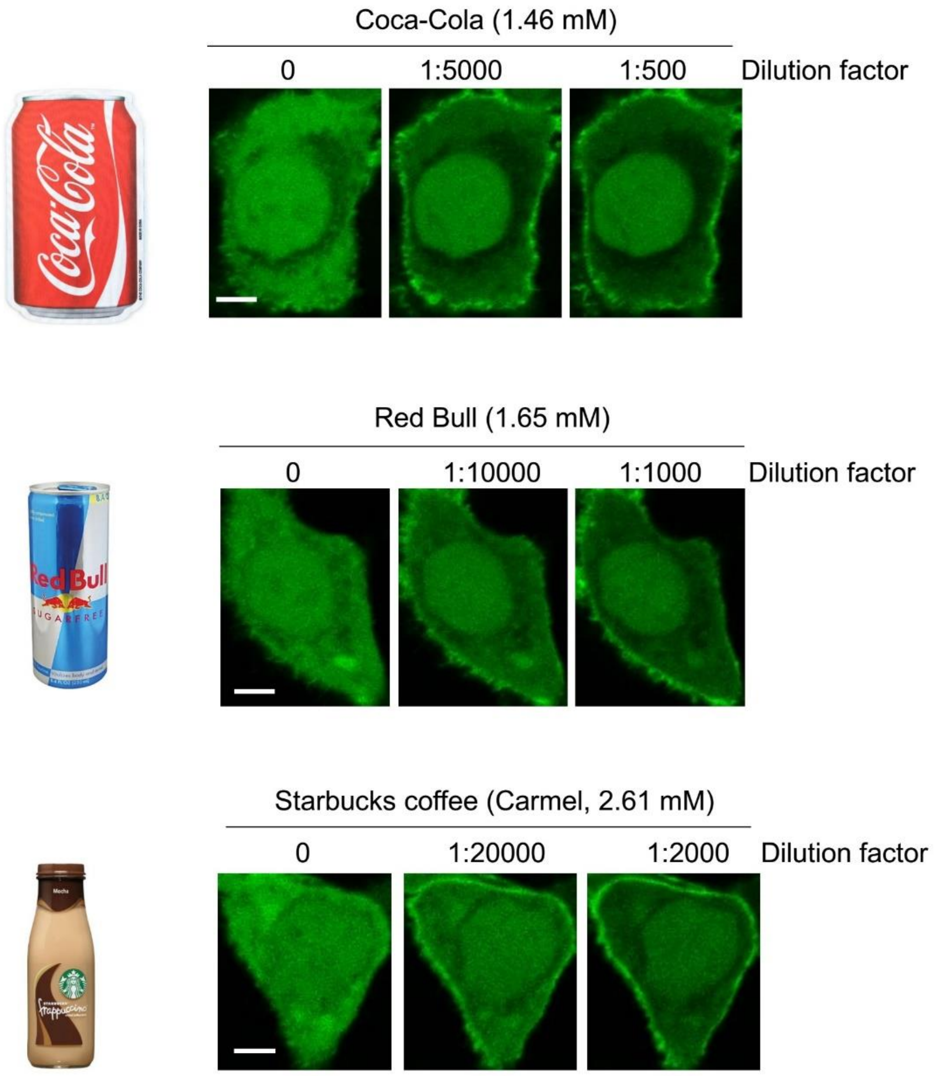


**Figure S6.** PM translocation of YFP-COSMO-PB induced by caffeinated beverages. Shown were representative confocal images of HeLa cells expressing YFP-COSMO-PB (green) before and after incubation with the indicated caffeine-containing beverages. The dilution factors were indicated above the images. Scale bar, 10 µm.

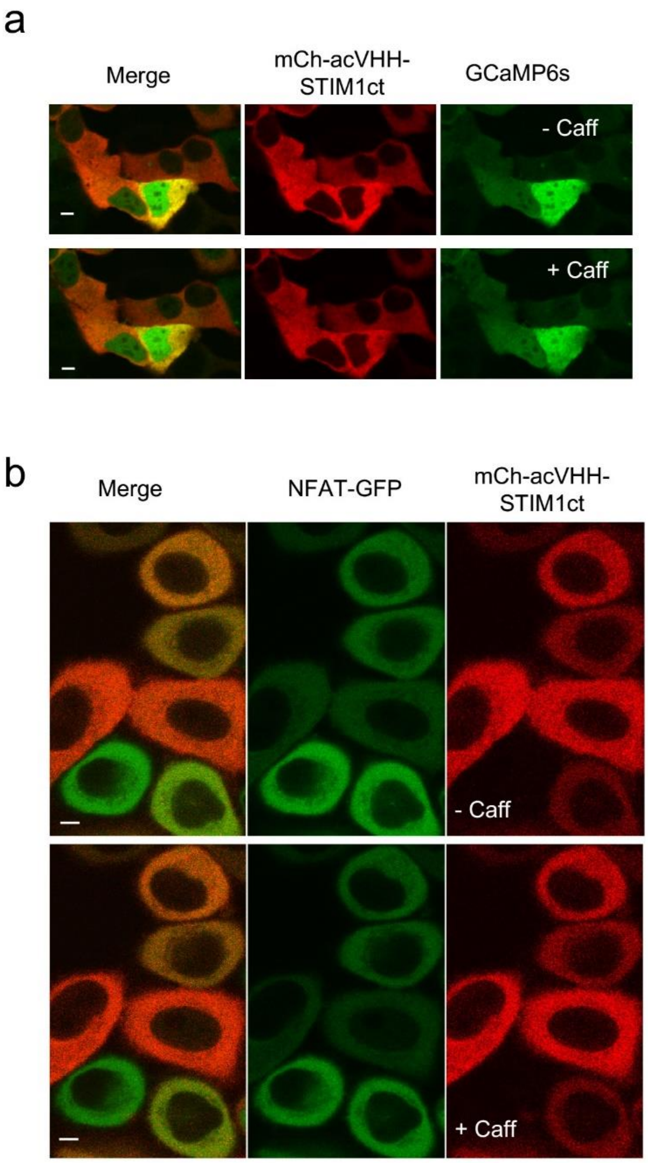


**Figure S7.** acVHH-STIM1ct failed to elicit $Ca^{2+}$ influx and NFAT nuclear entry following caffeine addition.
a) Confocal images of HeLa cells co-transfected with a green $Ca^{2+}$ sensor (GCaMP6s) and mCh-acVHH-STIM1ct (red) before and after 1 µM caffeine treatment for 10 min. Scale bar, 10 µm. b) Confocal images of HeLa-NFAT-GFP stable cells (green) transfected with mCh-acVHH-STIM1ct (red) before and after 1 µM caffeine treatment for 1 hour. The quantifications of $Ca^{2+}$ influx and NFAT nuclear translocation were shown in Figure 2e and 2f. Scale bar, 10 µm.

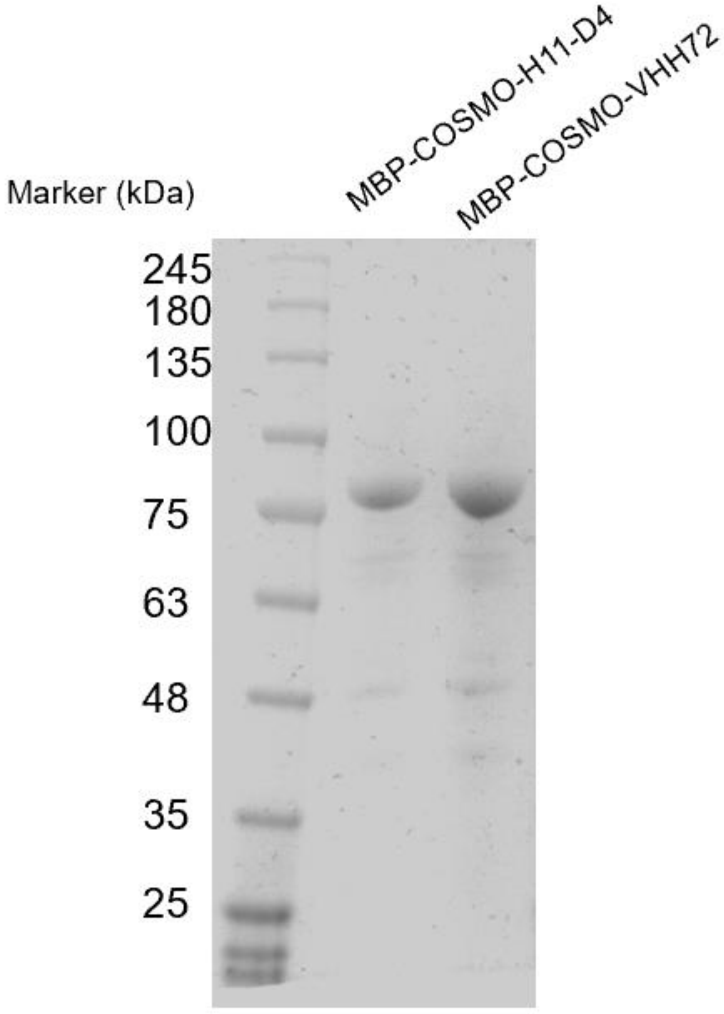


**Figure S8.** SDS-PAGE analysis of purified MBP-COSMO-H11-D4 and MBP-COSMO-VHH72 proteins. The calculated molecular weights of both proteins were approximately 76 kDa.

PM binding assay

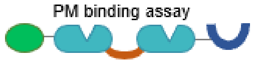


| Linker | Sequence | Structure | VHH (WT) PM binding -Caff | VHH (WT) PM binding + Caff | VHH (WT) EC50 (nM) | VHH (Y104W) PM binding -Caff | VHH (Y104W) PM binding + Caff | VHH (Y104W) EC50 (nM) | Binding models |
|---|---|---|---|---|---|---|---|---|---|
| 1 | GAP | flexible | – | +++ | 107 | + | ++++ | ND | Intermolecular dimerization 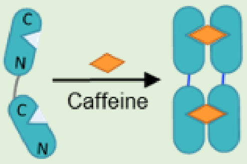 |
| 2 | DIGAP | flexible | – | +++ | 375 | + | ++++ | ND | |
| 3 | DIGSGAP | flexible | – | +++ | 61 | + | ++++ | ND | |
| 4 | DIGGSGGAP | flexible | – | +++ | 194 | + | ++++ | ND | |
| 5 | LHRAEQSLH DLGAP | rigid | – | +++ | 530 | – | ++++ | 16.9 | |
| 6 | HAAAGAPVP YPDPLEPRE QKLISEEDL GGSGGAP | rigid and flexible | – | – | ND | – | – | ND | Intramolecular dimerization 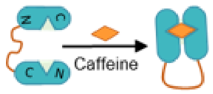 |



Linker 5: selected sequence from STIM1$_{L251-L261}$ (red region)

STIM1-CC1: LKMDLEGLHRAEQSLHDLQERLHKAQEE (PDB: 4O9B)

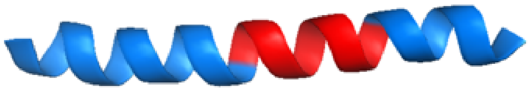

Linker 6: the predicated structure by TASSER

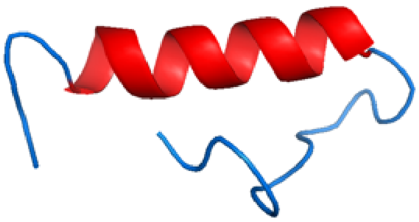

**Figure S9.** Linker optimization for biCOSMO-S/L. The top table showed the rationally designed linkers and their performance after caffeine treatment. The bottom panel showed the crystal structure of Linker 5 (PDB: 4O9B)[1] and the putative structure of Linker 6 predicted by I-TASSER[2].

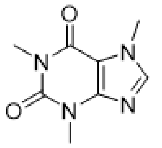

Caffeine

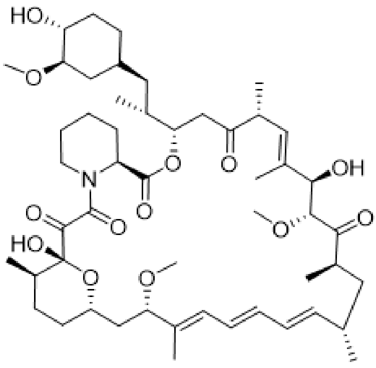

Rapamycin

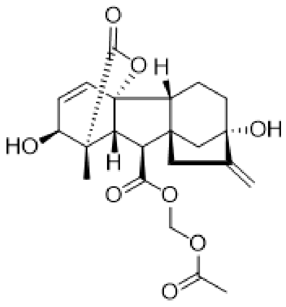

$GA_3$-AM

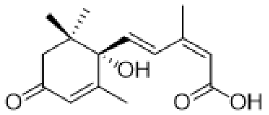

abscisic acid (ABA)

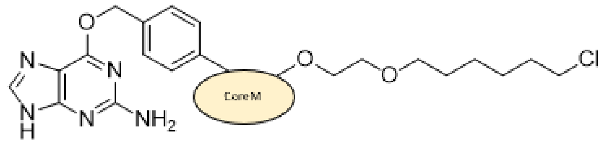

HaXS

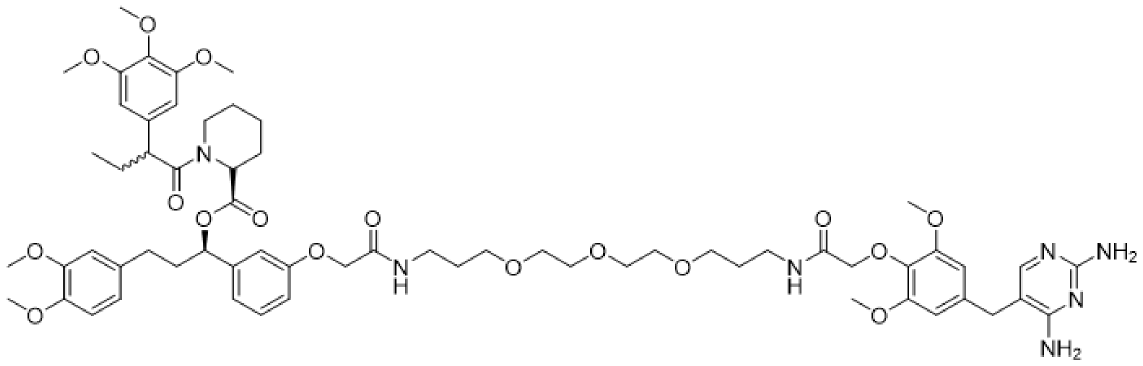

SLF’-TMP

**Figure S10.** Chemical structures of ligands used in CID systems.

**Table S1.** Summary of properties of current CID systems.

| Name | $EC_{50}$ (nM) | Kinetics (dimerization) | Ligands Price (from sigma) | Safety of ligands | References |
|---|---|---|---|---|---|
| COSMO/ Caffeine | 95.1 | < 1 min | $40.9 / 100 g | Daily beverage < 400 mg/day, adult | This study |
| biCOSMO-S/ Caffeine | 16.9 | < 1 min | $40.9 / 100 g | Daily beverage < 400 mg/day, adult | This study |
| FRB-FKBP/ Rapamycin | 10-20 | < 2 min | $407 / 5 mg | Brand name Sirolimus, as an immunosuppressive agent in clinics. Common side effects | 3, 4 |
| GAI-GID1 /GA3-AM | 310 | < 1 min | $256 / 5 mg | Relatively harmless for animals and human | 5 |
| PYL-ABI/ ABA | > 104 | > 30 min | $26.8 / 50 mg | Orally available | 6 |
| HaloTag-SNAPTag/ HaXS | > 103 | > 10 min | Not commercially available | Unknown | 7 |
| FKBP-eDHFR/ SLF-TMP | 120 | < 2 min | Not commercially available | No cytotoxicity toward COS-7 cells up to a concentration of 50 mM | 8 |

**Supplementary References**


[1] B. Cui, X. Yang, S. Li, Z. Lin, Z. Wang, C. Dong, Y. Shen, *PLoS One* **2013**, *8*, e74735.

[2] J. Yang, R. Yan, A. Roy, D. Xu, J. Poisson, Y. Zhang, *Nat. Methods* **2015**, *12*, 7.

[3] L. A. Banaszynski, Corey. W. Liu, Thomas. J. Wandless, *J. Am. Chem. Soc.* **2005**, *127*, 4715.

[4] M. V. Blagosklonny, *Aging* **2019**, *11*, 8048.

[5] T. Miyamoto, R. DeRose, A. Suarez, T. Ueno, M. Chen, T. P. Sun, M. J. Wolfgang, C. Mukherjee, D. J. Meyers, T. Inoue, *Nat. Chem. Biol.* **2012**, *8*, 465.

[6] F. Liang, W. Q. Ho, G. R. Crabtree, *Sci Signal.* **2011**, *164*, rs2.

[7] D. Erhart, M. Zimmermann, O. Jacques, M. B. Wittwer, B. Ernst, E. Constable, M. Zvelebil, F. Beaufils, M. P. Wymann, *Chem. Biol.* **2013**, *20*, 549.

[8] P. Liu, A. Calderon, G. Konstantinidis, J. Hou, S. Voss, X. Chen, F. Li, S. Banerjee, J. E. Hoffmann, C. Theiss, L. Dehmelt, Y. W. Wu, *Angew. Chem. Int. Ed. Engl.* **2014**, *53*, 10049.

**Captions for Supplementary Movies S1-4**

**Movie S1.** Time-lapse confocal imaging of HeLa cells expressing YFP-acVHH-PB in response to addition of 1 µM caffeine (0 sec-215 sec), wash-out (215 sec-1035 sec), and re-addition of 1 µM caffeine (1035 sec-1270 sec). The caffeine induced effect was fully reversible.

**Movie S2.** In-cellulo titration of YFP-COSMO-PB with escalating concentrations of caffeine for quantitative measurement of its $EC_{50}$ (0, 1, 3, 9, 27, 81, 243, 729, 2187, and 6561 nM).

**Movie S3.** Comparison of YFP-biCOSMO-S-PB (left) and YFP-biCOSMO-L-PB (right) upon in-cellulo titration with caffeine (0, 1, 2, 3, 9, 27, 81, 243, 729, 2187, 6561, and 19683 nM).

**Movie S4.** Time-lapse confocal imaging of HeLa cells expressing $MLKL_{NT}$-mCh-biCOSMO-S following the addition of 1 µM caffeine. Scale bar, 10 µm.
